# Multi-rate Poisson tree processes for single-locus species delimitation under maximum likelihood and Markov chain Monte Carlo

**DOI:** 10.1093/bioinformatics/btx025

**Published:** 2017-01-20

**Authors:** P Kapli, S Lutteropp, J Zhang, K Kobert, P Pavlidis, A Stamatakis, T Flouri

**Affiliations:** 1 The Exelixis Lab, Scientific Computing Group, Heidelberg Institute for Theoretical Studies, Heidelberg, Germany; 2 Department of Informatics, Institute of Theoretical Informatics, Karlsruhe Institute of Technology, Karlsruhe, Germany; 3 Foundation for Research and Technology, Hellas Institute of Computer Science GR, Heraklion, Crete, Greece

## Abstract

**Motivation:**

In recent years, molecular species delimitation has become a routine approach for quantifying and classifying biodiversity. Barcoding methods are of particular importance in large-scale surveys as they promote fast species discovery and biodiversity estimates. Among those, distance-based methods are the most common choice as they scale well with large datasets; however, they are sensitive to similarity threshold parameters and they ignore evolutionary relationships. The recently introduced “Poisson Tree Processes” (PTP) method is a phylogeny-aware approach that does not rely on such thresholds. Yet, two weaknesses of PTP impact its accuracy and practicality when applied to large datasets; it does not account for divergent intraspecific variation and is slow for a large number of sequences.

**Results:**

We introduce the multi-rate PTP (mPTP), an improved method that alleviates the theoretical and technical shortcomings of PTP. It incorporates different levels of intraspecific genetic diversity deriving from differences in either the evolutionary history or sampling of each species. Results on empirical data suggest that mPTP is superior to PTP and popular distance-based methods as it, consistently yields more accurate delimitations with respect to the taxonomy (i.e., identifies more taxonomic species, infers species numbers closer to the taxonomy). Moreover, mPTP does not require any similarity threshold as input. The novel dynamic programming algorithm attains a speedup of at least five orders of magnitude compared to PTP, allowing it to delimit species in large (meta-) barcoding data. In addition, Markov Chain Monte Carlo sampling provides a comprehensive evaluation of the inferred delimitation in just a few seconds for millions of steps, independently of tree size.

**Availability and Implementation:**

mPTP is implemented in C and is available for download at http://github.com/Pas-Kapli/mptp under the GNU Affero 3 license. A web-service is available at http://mptp.h-its.org.

**Supplementary information:**

Supplementary data are available at *Bioinformatics* online.

## 1 Introduction

Species are fundamental units of life and form the most common basis of comparison in evolutionary studies. Therefore, species delimitation is a critical task in systematic studies with potential implications in all subfields of biology that involve evolutionary relationships. In line with the species concept of De Queiroz (2007) and the integrative taxonomy approach (Dayrat, 2005), a reliable identification of a new species involves data from multiple sources (e.g., ecology, morphology, evolutionary history). Such an approach is necessary for meticulous comparative evolutionary studies but is tremendously difficult to apply, if at all possible, in large biodiversity studies (e.g., vast barcoding, environmental, microbial samples). In such studies, researchers use alternative units of comparison that are easier to delimit [Molecular Operational Taxonomic Units (Blaxter *et al.*, 2005), Recognizable Taxonomic Units (Oliver and Beattie, 1993)] rather than going through the cumbersome task of delimiting and describing full species. The correspondence of these units to real species remains ambiguous (Casiraghi *et al.*, 2010), especially in the absence of additional information. However, they are practical for biodiversity and *β*-diversity estimates (Valentini *et al.*, 2009).

With the introduction of DNA-barcoding (Hebert *et al.*, 2003) and the advances in coalescent models (Fujisawa and Barraclough, 2013; Yang and Rannala, 2014), genetic data became the most popular data source for delimiting species. Several algorithms and implementations exist for this purpose, most of which are inspired by the phylogenetic species concept (Fujisawa and Barraclough, 2013; Yang and Rannala, 2014; Zhang *et al.*, 2013) and the DNA barcoding concept (Hao *et al.*, 2011; Edgar, 2010; Puillandre *et al.*, 2012). These methods address different research questions (Casiraghi *et al.*, 2010); thus, the user needs to assess several factors before choosing the most appropriate method for a particular study. The “species-tree” approaches rely on multiple genetic loci (Jones *et al.*, 2015; Yang and Rannala, 2014) and account for potential species-tree/gene-tree incongruence (Maddison, 1997). Such methods are the most appropriate when the goal is to perform taxonomic revisions. However, most current implementations (Jones *et al.*, 2015; Yang and Rannala, 2014) are computationally demanding and can only be applied to small datasets of closely related taxa, becoming impractical with a growing number of samples and/or loci [see Fujisawa *et al.* (2016) for a recently introduced faster method]. Large-scale biodiversity (meta-) barcoding studies comprise hundreds or even thousands of samples of high evolutionary divergence. The goal of such studies often is to obtain β-diversity estimates (comparative studies of different treatments, ecological factors, etc.) or a rough estimate of the biodiversity for a given sample. Hence, a researcher may use distance-based methods (Edgar, 2010; Hao *et al.*, 2011; Puillandre *et al.*, 2012) that scale well on large datasets with respect to run times. However, these methods ignore the evolutionary relationships of the involved taxa and rely on not necessarily biologically meaningful *ad hoc* sequence similarity thresholds. Moreover, they are restricted to single-locus data, which reduces the accuracy of the delimitation (Dupuis *et al.*, 2012).

The General Mixed Yule Coalescent (GMYC; Fujisawa and Barraclough, 2013; Pons *et al.*, 2006) and the recently introduced Poisson Tree Processes (PTP; Zhang *et al.*, 2013) are two similar models that bridge the gap between “species-tree” and distance-based methods. While GMYC and PTP are also restricted to single loci, they do take the evolutionary relationships of the sequences into account. Since, at the same time, they are computationally inexpensive they can be deployed for analyzing large (meta-)barcoding samples. The GMYC method (Fujisawa and Barraclough, 2013) uses a speciation (Yule, 1925) and a neutral coalescent model (Hudson, 1990). It strives to maximize the likelihood score by separating/classifying the branches of an ultrametric tree (in units of absolute or relative ages) into two processes; within and between species. In contrast to GMYC, PTP models the branching processes based on the number of accumulated expected substitutions between subsequent speciation events. PTP tries to determine the transition point from a between- to a within-species process by assuming that a two parameter model—one parameter for the speciation and one for the coalescent process—best fits the data. The underlying assumption is that each substitution has a small probability of generating a branching event. Within species, branching events will be frequent whereas among species they will be more sparse. The probability of observing *n* speciations for *k* substitutions follows a Poisson process and therefore, the number of substitutions until the next speciation event can be modeled via an exponential distribution (Zhang *et al.*, 2013). Given that PTP directly uses substitutions, it does not require an ultrametric input tree, the inference of which can be time consuming and error prone. Thus, PTP often yields more accurate delimitations than GMYC (Tang *et al.*, 2014).

Here, we introduce a new algorithm and an improved model as well as implementation of PTP that alleviates previous shortcomings of the method. The initial PTP assumes one exponential distribution for the speciation events and only one for the coalescent events, across all species in the phylogeny. While the speciation rate can be assumed to be constant among closely related species, the intraspecific coalescent rate and consequently the genetic diversity may vary significantly even among sister species. This divergence in intraspecific variation can be attributed to factors such as population size and structure, population bottlenecks, selection, life cycle and mating systems (see Bazin et al. (2006) for further details). Additionally, sampling bias may also be responsible for observing different levels of intraspecific genetic diversity and it is already known to decrease the accuracy of PTP (Zhang *et al.*, 2013). To incorporate the potential divergence in intraspecific diversity, we propose the novel multi-rate PTP (mPTP) model. In contrast to PTP, it fits the branching events of *each* delimited species to a *distinct* exponential distribution. Thereby it can better accommodate the sampling- and population-specific characteristics of a broader range of empirical datasets. In addition, we develop and present a novel, dynamic-programming algorithm for the mPTP model. The implementation is several orders of magnitude faster than the original PTP and yields more accurate delimitations (almost instantly) on large datasets comprising thousands of taxa. Note that, the original PTP requires days of computation time to analyze such datasets. Finally, we provide a *Markov chain Monte Carlo* (MCMC) sampling approach that allows for inferring delimitation support values.

## 2 Methods

The mPTP method takes as input a binary, rooted phylogenetic tree inferred with software such as RAxML (Stamatakis, 2014) or MrBayes (Ronquist *et al.*, 2012). Such software often infer unrooted trees; therefore, mPTP provides the option to root the input tree prior to the analysis either (a) implicitly on its longest terminal branch or (b) explicitly by providing an outgroup. In this section, we introduce the basic notation that is used throughout the manuscript, and provide a detailed description of the mPTP algorithm including the MCMC sampling method.

A binary rooted tree T=(V,E) is a connected acyclic graph where *V* is the set of nodes and *E* the set of branches (or *edges*), such that E⊂V×V. Each *inner node u* has a *degree* (number of branches for which *u* is an end-point) of 3 with the exception of the root node which has degree 2, while *leaves* (or *tips*) have degree 1. We use the notation (u,v)∈E to denote a branch with end-points u,v∈V, and ℓ:V×V→ℝ to denote the associated branch length. Finally, we use *T_u_* to denote the subtree rooted at node *u*.

### 2.1 mPTP heuristic algorithm

Let T=(V,E) be a binary rooted phylogenetic tree with root node *r*. The optimization problem in the original PTP is to find a connected subgraph $G=(V_{s},E_{s})$ of *T*, where $V_{s}\subseteq V,\;E_{s}\subseteq E,\;r\in V_{s}$ such that (a) *G* is a binary tree, and (b) the likelihood of i.i.d branch lengths *E_s_* and $E_{c}=E\setminus E_{s}$ fitting two distinct exponential distributions is maximized. Formally, we are interested in maximizing the likelihood

(1) $$L=\left(\prod_{x\in E_{s}}{\hat{\lambda }}_{s}e^{-{\hat{\lambda }}_{s}\ell (x)}\right)\times \left(\prod_{x\in E_{c}}{\hat{\lambda }}_{c}e^{-{\hat{\lambda }}_{c}\ell (x)}\right)$$

where ${\hat{\lambda }}_{s}={\left(\frac{1}{\left|E_{s}\right|}\sum_{x\in E_{s}}\ell (x)\right)}^{-1}$ and ${\hat{\lambda }}_{c}={\left(\frac{1}{\left|E_{c}\right|}\sum_{x\in E_{c}}\ell (x)\right)}^{-1}$ are maximum-likelihood (ML) estimates for the rate parameters.

In the mPTP, we are interested in fitting the branch lengths of *each* maximal subtree of *T* (each species delimited in *T*) formed by branches from *E_c_* to a *distinct* exponential distribution. Let $T_{1}=(V_{1},E_{1}),T_{2}=(V_{2},E_{2}),\ldots ,T_{k}=(V_{k},E_{k})$ be the *maximal* subtrees of *T* formed exclusively by branches from *E_c_* such that ${⊔}_{i=1}^{k}E_{i}=E_{c}$ and no pair *i*, *j* exists for which $V_{i}\cap V_{j}\neq \emptyset$. The task in mPTP is to maximize the likelihood

(2) $$L=\left(\prod_{i=1}^{k}\prod_{x\in E_{i}}{\hat{\lambda }}_{i}e^{-{\hat{\lambda }}_{i}\ell (x)}\right)\times \left(\prod_{x\in E_{s}}{\hat{\lambda }}_{s}e^{-{\hat{\lambda }}_{s}\ell (x)}\right)$$

where ${\hat{\lambda }}_{i}={\left(\frac{1}{\left|E_{i}\right|}\sum_{x\in E_{i}}\ell (x)\right)}^{-1}$ for 1≤i≤k. We refer to the *k* maximal subtree root nodes as *coalescent roots*.

Whether a polynomial-time algorithm exists to solve the two problems remains an open question. We assume that the problem is hard, and thus, we propose a greedy, dynamic-programming (DP) algorithm to solve it.

The algorithm visits all inner nodes of *T* in a bottom-up post-order traversal. For each node *u*, the DP computes an array of $|E_{u}|+1$ scores, where *E_u_* is the set of branches of subtree *T_u_*. Entry $0\leq i\leq |E_{u}|$ assumes that *T_u_* contains *i* speciation branches. For the case *i* = 0, *T_u_* is part of *a* coalescent process, while for $i=|E_{u}|$, *T_u_* is part of *the* single speciation process. Each entry *i* > 0 contains the maximization of Equation (2), by considering only (i) the branches of subtree *T_u_* and (ii) the set *S* of branches of *T* that have at least one end-point on the path from the root *r* to *u*. For this subset of branches, the speciation process consists of exactly |S|+i branches, and we only consider the coalescent processes inside *T_u_*. We restrict ourselves to the set of branches *S* since it is infeasible to test all possible groupings of branches outside of *T_u_*. For any *i* > 0, it holds that the edges from *u* leading to its child nodes *v* and *w* belong to the speciation process, and, therefore, *S* is the smallest set of edges which, by definition, must be part of the speciation process.

**Fig. 1.**
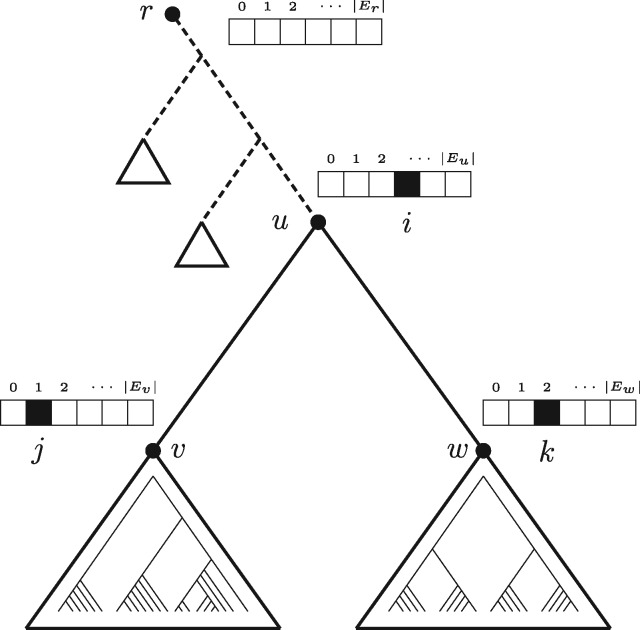
Visual representation of the mPTP dynamic programming algorithm. Each entry *i* at node *u* is computed from information stored at entries *j* and *k* of child nodes *v* and *w*, for all *j* and *k* such that i=j+k+2. The dashed branches denote the smallest set *S* of branches which, by definition, must be part of the speciation process, irrespective of the resolution of other subtrees outside *T_u_*

Figure 1 illustrates the algorithm for a triplet of nodes *u*, *v*, *w*, and marks the set *S* with dashed lines. Entry 0 represents the null-model for *T_u_*, that is, all branches of *T_u_* belong to the coalescent process [or, equivalently, to the speciation process, as the two cases can not be distinguished by the (m)PTP model]. The score (maximization of Equation (2)) for entry i≥1 is computed from the information stored in the array entries *j* and *k* of the two child nodes *v* and *w*, by considering all combinations of *j* and *k* such that i=j+k+2 (plus two accounts for the two outgoing edges of *u*). Not all array entries are necessarily valid. For instance, entry *i* = 1 may be invalid given that node *u* has two out-going branches in the speciation process. Each entry *i* at node *u* stores the computed score, the sum of the speciation branch lengths $\sigma_{u}^{i}$ within *T_u_*, the product $L_{u}^{i}$ of likelihoods of coalescent processes inside *T_u_*, and pointers for storing which entries *j* and *k* were chosen for calculating a specific entry *i*. The first term of Equation (2) (coalescent process) is the product of $L_{v}^{j}$ and $L_{w}^{k}$, while the second term (speciation process) is computed from the sums $\sigma_{v}^{j}$ and $\sigma_{w}^{k}$ and the sum of branch lengths from *S*. We store the score and child node entry indices for the combination of *j* and *k* that maximize the score.

Once the root node of *T* is processed, we have the likelihood values of exactly |E|+1 delimitations, one of which corresponds to the null-model (i=0). We select the entry *i* that minimizes the *Akaike Information Criterion* score corrected for small sample size (${\text{AIC}}_{c}$; McQuarrie and Tsai, 1998). This way we penalize over-splitting caused by the increasing number of parameters ($\lambda_{1},\lambda_{2},\ldots ,\lambda_{k}$). A lower ${\text{AIC}}_{c}$ value corresponds to a model which better explains the data. Once the best ${\text{AIC}}_{c}$ corrected entry *i* at the root has been determined, we perform a backtracking procedure using the stored child node pointers to retrieve the coalescent roots and hence obtain the species delimitation. The average asymptotic run-time complexity of the method is $O(n^{2})$ with a worst-case of $O(n^{3})$, where *n* is the number of nodes in *T* (for the proofs see Paragraph 1, Supplementary Material SI).

### 2.2 MCMC sampling

One way to assess the confidence of the best-scoring delimitation is to iterate through the finite set Ω of all possible delimitations, and compute a support value for every node *u* of the tree. This value is the sum of Akaike weights (Akaike, 1978; Burnham and Anderson, 2002) for all delimitations where *u* is part of the speciation process. Since this is infeasible for large trees, we deploy a discrete-time MCMC approach to approximate the probability distribution *π* on Ω that assigns an Akaike weight to each possible delimitation. Hence, by sampling according to the distribution *π*, we are able to approximate the support value for each node of the tree. At each step, we propose a new delimitation by designating a set of coalescent roots and compute its score. We denote the current delimitation as *θ* and the proposed delimitation, after applying a move to *θ*, as θ′. We use the acceptance ratio

$$\alpha =\frac{L_{c}^{\prime }}{L_{c}}\times \frac{g(\theta \prime \to \theta )}{g(\theta \to \theta \prime )}$$

to decide whether to keep the proposed delimitation θ′ or not. We assume that all delimitations are equally probable, and, therefore we use a uniform prior (cancelled out). If α≥1, we accept the delimitation, otherwise we accept it with probability *α*. The term $L_{c}^{\prime }/L_{c}$ is the ratio of the ${\text{AIC}}_{c}$-corrected likelihood values of θ′ over *θ*, and g(x→y) is the transition probability from one delimitation, *x*, to any other delimitation *y*. Given *θ*, we propose θ′ by applying one of two possible moves with equal probability. One of the two moves selects a coalescent process at random and splits it into two new coalescent processes, by placing its root node (and its two out-going branches) into the speciation process. The other type of move performs the reverse; a randomly selected node *u*, whose two child nodes are coalescent roots, is removed from the speciation process, effectively merging the two coalescent processes into a new one. The method’s run-time is linear to the number of MCMC steps and independent of the tree size. A full description of the MCMC algorithm appears in Paragraph 3 of Supplementary Material SI.

It is considered good practice that MCMC results are accompanied by a critical convergence assessment. Arguably, a good way of accomplishing this is to compare samples obtained from independent MCMC runs. We use the average standard deviation of delimitation support values (ASDDSV) for quantifying the similarity among such samples. Inspired by the average standard deviation of split frequencies (ASDSF) technique from phylogenetic inference (Ronquist *et al.*, 2012), we calculate the ASDDSV by averaging the standard deviation of per-node delimitation support values across multiple independent MCMC runs that explore the delimitation space. Each such MCMC run starts from a randomly generated delimitation. Similarly to ASDSF, ASDDSV approaches zero as runs converge to the same distribution of delimitations.

ASDDSV is useful for monitoring that runs converge to the same distribution. To further assess the agreement between the sampled delimitations and the best scoring delimitation on large datasets, where visual inspection is impractical, we introduce the *average support value* (ASV), that is,

$$\frac{1}{\left|V_{T}|+|V_{R}\right|}\left(\sum_{u\in V_{T}}f(u)+\sum_{u\in V_{R}}1-f(u)\right),$$

where *V_T_* is the set of tip nodes within the speciation process, *V_R_* is the set of coalescent roots, and *f*(*u*) the support value of node *u*. The closer ASV is to one, the better the support values agree with the ML delimitation.

## 3 Experimental setup

For the evaluation of mPTP, we use empirical datasets that comprise a number of varying parameters (substitution rate, geographic range, genetic diversity etc.), which often occur in practice and are difficult to reproduce with simulations. We retrieved all datasets from the Barcode of Life Database (BOLD, http://www.boldsystems.org/), the largest barcode library for eukaryotes. For each of the datasets, we inferred the putative species with mPTP and four other methods that model speciation on the basis of genetic distance (including the original PTP method), and assess their accuracy with respect to the current taxonomy (available in BOLD). Finally, we avoided comparisons with time-based delimitation methods (Fujisawa and Barraclough, 2013; Jones *et al.*, 2015; Yang and Rannala, 2014) which are time consuming and heavily dependent on the factorization accuracy of branch lengths into time and evolutionary rate.

### 3.1 Empirical datasets

We retrieved 24 empirical genus level datasets (Table 1) that cover six of the most species rich animal Phyla in BOLD (Arthropoda, Annelida, Chordata, Echinodermata, Platyhelminthes and Cnidaria). This allows us to evaluate the efficiency of mPTP given a wide range of organisms with diverse biological traits and evolutionary histories. The majority of datasets (14 out of 24 datasets) belong to the Arthropoda which is the most species-rich animal group, and the most common group PTP has been applied to so far (in 61 out of 110 citations). Within Arthropoda, we mainly focus on insects (8 out 14 datasets), which represents over 90% of all animals. The rest of the empirical datasets spans the remaining five phyla.

**Table 1.** Main characteristics of empirical test datasets

Genus	Phylum	NSp	NMSp	NSeq	AL	APD (%)
*Amynthas*	Annelida	43	28	265	1539	16.7
*Phyllotreta*	Arthropoda	16	14	195	659	11.8
*Atheta*	Arthropoda	84	60	399	1076	14.2
*Philodromus*	Arthropoda	15	11	493	713	10.2
*Digrammia*	Arthropoda	20	17	286	659	6.2
*Carabus*	Arthropoda	49	34	514	880	11.5
*Bembidion*	Arthropoda	97	80	532	1495	12.9
*Calcinus*	Arthropoda	29	24	641	679	16.6
*Xysticus*	Arthropoda	35	33	861	1238	8.9
*Gammarus*	Arthropoda	53	27	755	1082	21.4
*Clubiona*	Arthropoda	35	32	1127	1256	8.4
*Culicoides*	Arthropoda	100	76	1252	1485	17.5
*Balanus*	Arthropoda	4	3	1775	1548	2.3
*Drosophila*	Arthropoda	148	109	2303	1589	11.2
*Anopheles*	Arthropoda	121	68	2741	1544	10.5
*Anolis*	Chordata	41	35	181	709	18.9
*Coryphopterus*	Chordata	12	10	282	658	14.7
*Myotis*	Chordata	54	37	789	1542	11.7
*Cyanea*	Cnidaria	5	4	92	1002	12.7
*Ophiura*	Echinodermata	13	8	75	1605	16.8
*Holothuria*	Echinodermata	18	12	355	1553	13.6
*Mopalia*	Mollusca	20	11	304	708	10.9
*Rhagada*	Mollusca	24	12	686	675	11.1
*Echinococcus*	Platyhelminthes	7	5	316	1608	5.1

NSp, number of species; NMSp, number of monophyletic species; NSeq, number of sequences; AL, alignment length; APD, average P-distance.

All datasets comprise the 5′ end of the common animal barcoding gene “Cytochrome Oxidase subunit I” (COI-5P) (Hebert *et al.*, 2003). The number of taxonomic species in the 24 datasets ranged from four (*Balanus*) to 148 (*Drosophila*), while the number of sequences from 75 (*Ophiura*) to 2741 (*Anopheles*). The number of sequences per species ranged between two and the extreme case of 1763 for *Balanus glandula*, reflecting situations where some species might be readily and others rarely available. Finally, the geographical distribution of the datasets also varied from the global scale (e.g., *Anopheles* and *Drosophila*) to the local scale (e.g., the *Anolis* samples originate from the islands and surrounding shores of the Caribbean sea).

### 3.2 Putative species delimitation

The sequence files obtained from the BOLD database were preprocessed to remove three factors that interfere with the assessment of the delimitation methods. First, the efficiency of each method is measured by comparing the delimited to the taxonomic species (see Section 3.3). Therefore, it was necessary to remove sequences of ambiguous taxonomic assignment. Second, we removed duplicate sequences, using the “-f c” option of RAxML version 8.1.17 (Stamatakis, 2014), to avoid unnecessary computations. Finally, for all methods that infer the shifting point among speciation and coalescent based on the data (e.g., Puillandre *et al.*, 2012), it is important to assume adequate representation of both processes. Therefore, we removed singleton species that could be a potential source of error in this framework (i.e. the lack of multiple sequences per species will inevitably decrease the distinctiveness of the two evolutionary processes and consequently the identification of the shifting point from one to the other).

**Fig. 2.**
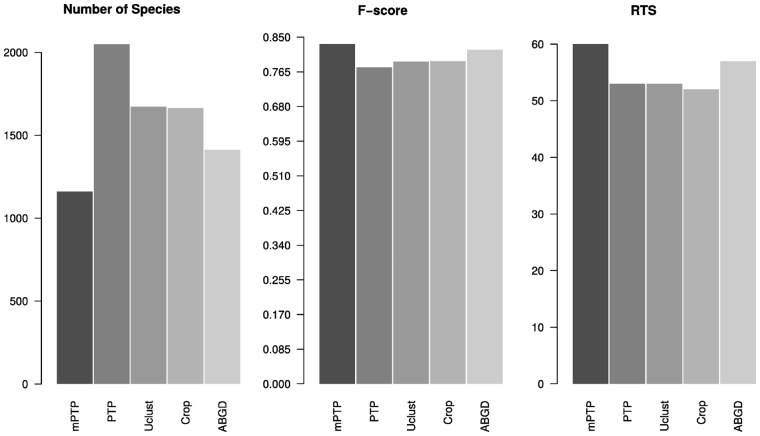
Average performance over all datasets of the five delimitation methods (mPTP, PTP, Usearch, Crop and ABGD) for the (A) number of species, (B) *F*-scores and (C) number of RTS

For each dataset, we inferred putative species using the two PTP models and we compared the results to three popular and well-established distance-based methods; ABGD (Puillandre *et al.*, 2012), Usearch (Edgar, 2010) and Crop (Hao *et al.*, 2011). The input format of the data and parameters differ among the five methods. The two PTP versions require a rooted phylogenetic tree. Therefore, we used Mafft v7.123 (Katoh and Standley, 2013) to align the sequences of each dataset, and subsequently RAxML under the default algorithm and the GTR + Γ model, to infer an ML tree. To root each tree, we chose outgroup taxa based on previously published phylogenies. For the genera without such prior phylogenetic knowledge, we selected a representative species of a genus belonging to the same family or tribe given the taxonomy in BOLD (NCBI Accession Numbers of the ingroup and the outgroup sequences are provided in Supplementary Material SII). Moreover, we implemented a method that identifies and ignores branches that result from identical sequences, during the delimitation process (for more details see Section 2 and Figs 1 and 2, Supplementary Material SI).

For mPTP, we further assessed the confidence of the ML solution using MCMC sampling. For each dataset, we executed ten MCMC runs of $2\times 10^{7}$ steps, each starting from an initial random delimitation. We assess whether the independent runs have converged by calculating the ASDDSV. To obtain an overall support for the ML estimate, we computed the mean ASV over all 10 independent runs.

For each of the remaining three methods (ABGD, Usearch and Crop), we optimized a set of performance-critical parameters and chose the delimitation that recovered the highest number of taxonomic species. ABGD requires two parameters: (i) the prior limit to intraspecific diversity (*P*) and (ii) a proxy for the minimum barcoding gap among the inter- and intraspecific genetic distances (*X*). We ran ABGD with the aligned sequences and 400 parameter combinations. For *P*, we sampled 100 values from the range 〈0.001,0.1〉 and for *X* we used the four values 0.05, 0.1, 0.15 and 0.2. The only critical parameter for Usearch is the fractional identity threshold (*id*) which defines the minimum similarity for the sequences of each cluster. We performed 50 consecutive runs, increasing the *id* value by 0.01 starting with 0.5. Finally, for Crop, we tried four combinations of the *l* and *u* parameters that correspond to similarity thresholds of 1%, 2%, 3%, and 5% as suggested by the authors (http://code.google.com/p/crop-tingchenlab/). Another critical parameter for Crop is *z*, which specifies the maximum number of sequences to consider after the so-called initial “split and merge”. We set *z* to 100 which is considered a reasonable value for full-length barcoding genes.

### 3.3 Comparison of delimitation methods

The evaluation of the five methods is based on three measures. First, the percentage of recovered taxonomic species (RTS), that is, the percentage of delimited species that match the “true species”. We consider the taxonomic species retrieved from BOLD to be the “true species”, and we deem the performance of the algorithm better when the number of matches to the taxonomic species is higher. Since we do not have the expertise to evaluate the taxonomy of each dataset, we could not assess the accuracy of each individual delimitation. However, by assuming that the closer a delimitation is to the current taxonomy the higher the probability to correspond to the real species, we gain some insight as to the relative accuracy among the different methods. The second measure is the *F*-score, also known as *F*-measure or *F*1-score (Rijsbergen, 1979), that is, the harmonic mean of *precision* and *recall*. In species delimitation, *precision* denotes the fraction of clustered sequences belonging to a single taxonomic species. The *recall* describes the fraction of sequences of a species that are clustered together. The *F*-score improves when decreasing (i) the number of species which are split into more than one groups and (ii) the number of taxonomic species lumped together into one group. The *F*-score ranges from 0 to 1, where 1 indicates a perfect agreement among two delimitations. Finally, the third measure is simply the number of delimited species.

The accuracy of either PTP model in recovering the true species depends on whether these taxa form monophyletic clades. Thus, for PTP and mPTP, we also compare their performance by applying the above measures for monophyletic taxonomic species only. Additionally, we quantify how the percentage of monophyletic species correlates to the percentage of RTS for the five delimitation methods we test. Finally, we test whether the most complex model (mPTP) fits the data significantly better than the simpler one (PTP), by performing a Likelihood Ratio Test (LRT). The LRT comparison among PTP and mPTP is only possible when their likelihood scores are calculated on identical delimitations, in which case the PTP is nested in the mPTP delimitation. Such a comparison informs us whether the additional parameters in mPTP improve significantly the likelihood of the delimitation.

## 4 Results

We analyzed a total of 17 219 COI sequences. The alignment length ranged from 658 bp to 1620 bp, while the proportion of variable nucleotides, measured by average P-Distance (MEGA v5.2; Tamura *et al.*, 2011) for each dataset, varied from 2.3% (*Balanus*) to 21.4% (*Gammarus*). Table 1 presents the fraction of monophyletic taxonomic species in the phylogenies for each dataset. The fraction ranges from only 51% in *Gammarus*, the most variable of the datasets, to 94% (*Xysticus*). Out of the 400 combinations of parameters tested for ABGD, two recovered the highest number of species, with *P* = 0.010235 and *X* = 0.1 or 0.05. For Usearch, the delimitation that maximized the number of RTS was with the threshold value of *id* = 0.97. Finally, the best-scoring parameter combination for Crop (*l* = 1.0 and *u* = 1.5) corresponds to the 3% similarity threshold. Table 2 presents the efficiency of the five delimitation methods for five of the datasets using three measures: percentage of RTS, *F*-score and number of species. A complete list of results for all 24 datasets is given in paragraph 4.1 in Supplementary Material SI. Overall, mPTP and ABGD scored best in terms of RTS percentage (59% and 57% on an average, respectively) and *F*-scores (0.828 and 0.819, respectively) compared with the other three methods (PTP: RTS = 53%, *F*-score = 0.776, Usearch: RTS = 53%, *F*-score = 0.79, Crop: RTS = 52%, *F*-score = 0.791) (Fig. 2). The striking difference between the five methods is in the number of delimited species. The novel mPTP method delimited a total of 1190 species which is the closest to the total number of taxonomic species (1041). In contrast, PTP inferred 2048 species which is almost twice this number. The other three methods yielded more conservative species numbers compared to PTP (Usearch: 1671, Crop: 1663, ABGD: 1412) that are, however, still notably higher than the mPTP estimates (Fig. 2). In terms of likelihood, when we compared mPTP with PTP for a fixed delimitation, mPTP was always yielding at least as good scores as the PTP but higher in most cases (Supplementary Table S3 in Supplement I). Based on the LRT results the increase in the likelihood was significant in all datasets except three for which both models resulted in identical or very similar scores.

**Table 2.** Percentage of RTS, *F*-scores and number of delimited species for the five delimitation methods (mPTP, PTP, Usearch, Crop and ABGD) for five of the empirical datasets

	mPTP	PTP	Usearch	Crop	ABGD
Genus	**RTS (%)**				
*Amynthas*	51	40	44	40	44
*Anopheles*	41	39	30	31	40
*Atheta*	64	60	57	55	60
*Drosophila*	47	51	41	34	53
*Philodromus*	60	27	47	53	47
	**F-score**				
Amynthas	0.784	0.638	0.649	0.674	0.673
*Anopheles*	0.787	0.704	0.730	0.681	0.730
*Atheta*	0.839	0.844	0.836	0.832	0.850
*Drosophila*	0.728	0.559	0.747	0.729	0.765
*Philodromus*	0.882	0.717	0.812	0.852	0.828
	**Number of species**				
*Amynthas*	64	104	95	91	90
*Anopheles*	126	218	193	154	118
*Atheta*	85	100	95	95	91
*Drosophila*	139	444	183	217	157
*Philodromus*	21	38	26	20	24

**Fig. 3.**
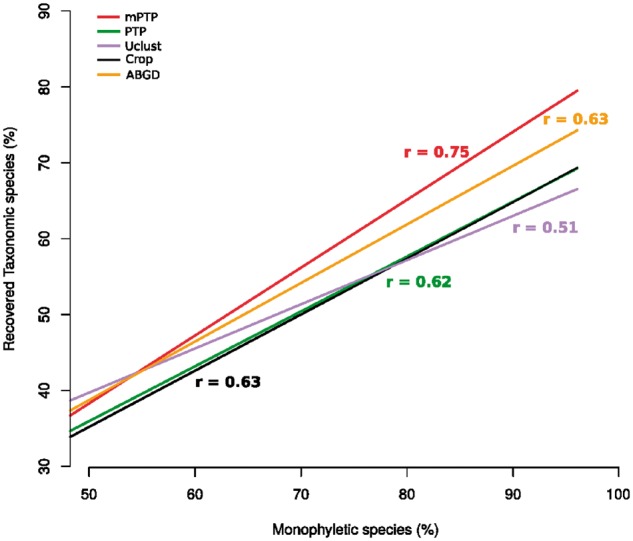
For each method, we fit a regression line to the points of correspondence of the percentage of monophyletic species (*x*-axis) to the percentage of RTS (*y*-axis). The Pearson coefficient (*r*) is given for each correlation in the corresponding color

When considering only monophyletic species, as one might expect, the recovery percentage increases substantially for both PTP models (82% and 72% on an average for mPTP and PTP, respectively). Similarly, the accuracy of the delimitations is substantially higher for the monophyletic taxonomic species (average *F*-score 0.945 and 0.853 for mPTP and PTP, respectively). These results indicate that polyphyly (we use the term polyphyly in referring to both paraphyly and polyphyly *sensu*, Funk and Omland, 2003) is a major contributing factor when taxonomic species are not recovered (Supplementary Table S2 in Supplement I). In particular, the RTS percentage of either PTP model is highly correlated with the percentage of monophyletic species in a dataset (Fig. 3). The Pearson coefficient indicates that the correlation is stronger among the species recovered with mPTP ($r_{mPTP}=0.75$, *P* value = 2.642e−05) than with PTP ($r_{PTP}=0.62$, *P* value = 0.001218). The correlation is also positive for the other three methods (*r_ABGD_* = 0.63/*P* value = 0.0009327, *r_Usearch_* = 0.5/*P* value = 0.01294, *r_Crop_* = 0.63/*P* value = 0.0009421) and comparable with PTP but smaller than for mPTP. Similarly, the slope of the regression line was greater for mPTP than for the other methods, indicating a steeper linear relationship between the two variables (Fig. 3).

Regarding the confidence of the mPTP delimitations, all independent MCMC runs appear to converge with an ASDDSV below 0.01 for all datasets, except for *Drosophila* (one of the largest datasets), for which the ASDDSV was 0.048, but still in the acceptable range for assuming convergence (Ronquist *et al.*, 2012). The ASV with respect to the ML delimitation was very high for all datasets, ranging from 72% (*Phyllotreta*) to 100% (*Balanus*), indicating that the data support well the ML solution (Supplementary Table S3 in Supplement I). The accumulated running time for all 10 independent runs (executed sequentially on an Intel Core i7-4500U CPU @ 1.80GHz) was less than 50 s on average across all datasets, which corresponds to ∼5 s per run. For a thorough run-time comparison between PTP and mPTP (including the ML method), refer to Paragraph 4.2 in Supplementary Material SI.

## 5 Discussion

Molecular species delimitation has caused mixed reactions within the scientific community, from those highly enthusiastic about its potential to accelerate biodiversity cataloging (Blaxter, 2003; Blaxter and Floyd, 2003) to those very critical about its role in shaping modern systematics (Bauer *et al.*, 2010; Will *et al.*, 2005). The main argument between the two conflicting sides is whether molecular delimitation on its own is sufficient to justify taxonomic rearrangements (Will *et al.*, 2005). Integrative taxonomy alleviates this conflict as it, by definition, requires multiple levels of evidence taking into account various biodiversity characteristics of an organism to accept potential taxonomic changes. Within this framework, and in line with the independently evolving species concept (De Queiroz, 2007) and the phylogenetic species concept, molecular species delimitation using DNA-barcoding serves as an excellent tool in modern taxonomy (Tautz *et al.*, 2003; Vogler and Monaghan, 2007). It can be easily applied to a large range of organisms regardless of their life stage, gender or prior taxonomic knowledge, by a broad range of researchers. In addition, barcoding genes are easily amplified from small tissue samples even from poorly preserved historical samples (Austin and Melville, 2006). Furthermore, such an approach might represent the only feasible approach in biodiversity surveys, as they often comprise a large number of species, many of which might be unknown or not easily accessible. Hence, collecting ecological or morphological traits is often simply not feasible. For historical samples or samples from inaccessible areas (e.g., deep seas, deserts) barcoding methods are equally important, since the collection of life history traits might be similarly challenging. This and previous studies (Monaghan *et al.*, 2009; Esselstyn *et al.*, 2012; Ratnasingham and Hebert, 2013; Tang *et al.*, 2014) suggest that single-locus barcoding methods provide meaningful clusters, close to taxonomically acknowledged species. This makes them useful for approximate species estimation studies or when more thorough systematic research is practically impossible.

### 5.1 (m)PTP

The molecular systematics of the example taxa vary from well-studied [e.g., *Anopheles* (Harbach and Kitching, 2016), *Drosophila* (Obbard *et al.*, 2012)] to scarcely studied (e.g., *Xysticus*, *Clubiona*). They further differ in the number of species, number of sequences per species, geographic ranges, and nucleotide divergences. Despite these differences, mPTP outperforms PTP and yields substantially smaller putative species numbers as well as delimitations that are closer to the current taxonomy. The assumption of mPTP that per-species branch lengths can be fit to distinct exponential distributions increases flexibility and adjustability to more realistic datasets. The divergence of intraspecific diversity patterns may either be due to population traits and processes (Bazin *et al.*, 2006) or the uneven sampling of the species (e.g., a highly sampled species from a single location compared to a species represented by one sample from multiple locations). The latter is known to decrease PTP accuracy (Zhang *et al.*, 2013).

The accuracy of (m)PTP strongly correlates with the proportion of monophyletic species in the underlying phylogeny. Polyphyletic species will either be delimited into smaller groups or delimited with other, nested species. Therefore, the recovery rate of taxonomic species is significantly higher when only considering the monophyletic species in each dataset. Among the 24 datasets, the number of monophyletic species ranged from 51% to 94%. Thus, the lack of monophyly is the primary contributing factor for not recovering taxonomic species with either PTP model. The average observed monophyly in our arthropod (76.4%) and the remaining invertebrate (64.7%) datasets corresponds well to available estimates for the same groups (73.5% and 61.4%, respectively) based on a large number of empirical studies (Funk and Omland, 2003). The same study reports that the most common reasons for polyphyly are inaccurate taxonomy, incomplete lineage sorting, and retrogressive hybridization (Funk and Omland, 2003; McKay and Zink, 2010). All three effects could apply to the selected datasets. Nevertheless, only the latter two are relevant to the efficiency of the algorithm *per se*, while the accuracy of the taxonomy is only relevant when it is used as a reference measure. Polyphyletic species affect both PTP versions in the same way. The reason for the improved delimitation accuracy of mPTP over PTP on monophyletic species is that the former accommodates different degrees of intraspecific genetic diversity within a phylogeny.

#### 5.1.1 MCMC sampling

The drawback of an ML approach is that it only provides a point estimate and no information on model uncertainty. The confidence about a given solution has a substantial impact in drawing reasonable conclusions. Therefore, we provide an MCMC method for assessing the plausibility of the ML solution. In phylogenies comprising hundreds of taxa, it is hard to obtain an overall support for a particular delimitation hypothesis by visual inspection of the tree. To alleviate this problem, at the end of an MCMC run we calculate the ASV for the ML solution. In our experiments, the ML delimitations were highly supported by the ASVs for all datasets, pointing towards a unimodal likelihood surface for our model (Supplementary Table S3 in Supplement I). Low ASVs may be interpreted as low confidence for the given delimitation scheme, either because another (multi-modal likelihood surface) or no delimitation scheme (flat-likelihood surface) is well supported. This also indicates a poor fit of the data to the model. The execution times of the MCMC sampling are almost negligible, regardless of tree size, and, therefore, we can thoroughly sample the delimitation space even for phylogenies comprising thousands of taxa. For the large phylogenies of our study (i.e., *Drosophila*, *Anopheles*), the PTP implementation required over 30 h for the ML optimization alone, and would require days or even months for the estimation of support values. Instead, mPTP required less than a minute for both the ML optimization and the support value estimation.

### 5.2 Distance-based methods

Distance-based methods are easy to apply to large datasets as they need minimal preprocessing effort and computational time. Their major weakness is that they require either a threshold value or a combination of parameters associated with the threshold value, the sampling effort, or the search strategy. Empirical data show that certain similarity cut-offs (2–3%) correspond well to the species boundaries of several taxonomic groups (Hebert *et al.*, 2003, Hebert *et al.*, 2004; ; Smith *et al.*, 2005); however, these values are often far from optimal (Lin *et al.*, 2015). Selecting these parameters is not intuitive and they may only be evaluated *a posteriori* based on the expectations of the researcher. Furthermore, the empirical knowledge for threshold settings is tightly associated with barcoding genes, and it may not be as useful for other marker genes. Here, we optimized the parameters for three of the most popular distance methods (ABGD, Crop and Usearch) based on the RTS percentage. Despite our substantial effort to use optimal parameters for the given data, our results show that, on an average, mPTP performs better with respect to *F*-scores and RTS percentage. At the same time, it also delimited notably fewer species. ABGD accuracy was the closest to mPTP, while PTP, CROP, and Usearch performed notably worse. Besides accuracy, the greatest advantage of mPTP is that it consistently yields more accurate results without requiring the user to optimize any parameters/thresholds. Finally, in contrast to (m)PTP, distance methods ignore evolutionary relationships. Hence, there is no direct relation between monophyletic species and delimitation accuracy in similarity based tools. However, polyphyly often reflects recent speciation while reciprocal monophyly indicates that significant time since speciation has passed. Consequently, the barcoding gap should be less pronounced in datasets of recently diverged species. This justifies that the RTS fraction improves with the number of monophyletic species.

## 6 Conclusions

We presented mPTP, a novel approach for single-locus delimitation that consistently provides faster and more accurate species estimates than PTP and other popular delimitation methods. As PTP, mPTP is mainly designed for analyzing barcoding loci, but can potentially also be applied to entire organelle phylogenies (e.g. mitochondria, Qin *et al.*, 2015). In contrast to methods based on sequence similarity, mPTP does not require any similarity threshold or other user-defined parameter as input. The limitations of mPTP are associated with processes that cannot be detected in either single-gene phylogenies (incomplete lineage sorting, hybridization) or in recent speciation events. The novel dynamic programming delimitation algorithm reduces computation time to a minimum and allows for almost instantaneous species delimitation on phylogenies with thousands of taxa. The MCMC sampling provides support values for the delimited species based on millions (or even billions) of MCMC generations in just a few seconds on a modern computer. The mPTP tool is available both, as a standalone package, and as a web service.

## Supplementary Material

Supplementary Data
