# Supplementary material for: Multi-rate Poisson tree processes for single-locus species delimitation under maximum likelihood and Markov chain Monte Carlo
Source: Bioinformatics. 2017 Jan 20;33(11):1630–8. doi: 10.1093/bioinformatics/btx025 (PMC5447239; doi:10.1093/bioinformatics/btx025)
Supplement: Supplementary Data [file btx025_supp.zip › supplement-i.pdf]

Multi-rate Poisson Tree Processes for single-locus species delimitation under Maximum Likelihood and Markov Chain Monte Carlo.

Supplementary Material I

Paschalia Kapli[1], Sarah Lutteropp[1,2], Jiajie Zhang[1], Kassian Kobert[1], Pavlos Pavlidis[3], Alexandros Stamatakis[1,2], and Tomáš Flouri[1,2]

[1] Heidelberg Institute for Theoretical Studies, Germany
[2] Karlsruhe Institute of Technology, Institute for Theoretical Informatics, Germany
[3] Institute of Molecular Biology and Biotechnology (IMBB), Foundation of Research and Technology, Heraklion, Greece

1 Asymptotic time and space complexities

Here we prove the asymptotic time complexity of the heuristic algorithm that estimates the maximum-likelihood delimitation. This supplement follows the notation introduced in the main paper.

Theorem 1 (Complexity). *Given a rooted binary tree of $n+1$ leaves, the two algorithms (single- and multi-rate) have an asymptotic run-time complexity of $\mathcal{O}(n^2)$ on average, and $\mathcal{O}(n^3)$ in the worst-case.*

Proof. W.l.o.g, we first compute the asymptotic run-time complexity for updating the array of size $m = |T_u| + 1$ at some inner node u of the binary tree T (the root of subtree T_u). Let v and w be the two child nodes of u, and T_u the subtree rooted at u. For computing an arbitrary entry i of the array, the algorithm uses each entry $j \in \{0, 1, \ldots, i\}$ of the array at node v in combination with entry $k = i - j$ at node w. Processing each such triplet i, j, k requires a constant time $c = \mathcal{O}(1)$. Therefore, the asymptotic time complexity for computing all m entries of node u is

$$\sum_{i=0}^{m-1}\sum_{j=0}^{i-2} c = \sum_{i=0}^{m-1}(i-1)c = c\left(\sum_{i=1}^{m-1} i - m\right) = c\left(\frac{m(m-1)}{2} - m\right) = \mathcal{O}(m^2). \quad (1)$$

To analyze the average case complexity of the algorithm, we first estimate the average cost for updating the entries of a node u. Since it is proportional to the size of the subtree T_u, we first estimate the average size of a subtree over all possible binary trees with $n+1$ tips. The number of possible binary trees with $n+1$ tips is given by the n-th Catalan number [1]

$$C_n = \frac{1}{n+1}\binom{2n}{n}$$

whose asymptotic growth is

$$C_n \sim \frac{4^n}{n^{3/2}\sqrt{\pi}} \quad (2)$$

(see [2]). The sum of all subtree sizes for all possible binary trees of $n+1$ tip nodes is

$$2^{2n+1} - \binom{2n+1}{n+1} \sim 2^{2n+1}\left(1 - \frac{1}{\sqrt{n\pi}}\right) \quad (3)$$

(see [3] and [4], pp. 184-185). The average subtree size $\overline{s}_n$ is the ratio of the sum of all subtree sizes for all possible binary trees with $n+1$ tip nodes (Equation 3) over the number of all subtrees (Equation 2 multiplied by the number of inner nodes n)

$$\overline{s}_n \sim \frac{2^{2n+1}(1-\frac{1}{\sqrt{n\pi}})}{n\frac{4^n}{n^{3/2}\sqrt{\pi}}} \tag{4}$$

Simplifying equation 4, we obtain the asymptotic average subtree size

$$\overline{s}_n \sim \frac{2^{2n+1}(1-\frac{1}{\sqrt{n\pi}})}{n\frac{4^n}{n^{3/2}\sqrt{\pi}}} = \frac{\frac{2^{2n+1}(\sqrt{n\pi}-1)}{\sqrt{n\pi}}}{\frac{2^{2n}(2n+1)}{n\sqrt{n\pi}}} = 2\frac{\sqrt{n\pi}-1)}{\frac{2n+1}{n}} \sim \mathcal{O}(\sqrt{n}). \tag{5}$$

Now, assume T is a tree of $n+1$ tips, and as such, consists of exactly n inner nodes. The cost for processing an inner node u is $\mathcal{O}(m^2)$ where $m = |T_u| + 1$. From Equation 5, the subtree size is $\mathcal{O}(\sqrt{n})$ on average. Therefore, the number of operations required for the algorithm is $n \times \mathcal{O}(\sqrt{n}^2)$ on average, which equals to $\mathcal{O}(n^2)$.

For computing the worst-case time complexity, we first set an upper bound which we trivially know cannot be exceeded. We consider that if *all* subtrees of T were of size $\mathcal{O}(n)$, then the worst-case complexity would be $\mathcal{O}(n^3)$. This bound can be obtained in a similar way as the average-case time complexity. We show that this bound can be reached with *caterpillar* trees, i.e., trees in which all nodes are within distance 1 of a central path. Consider a caterpillar tree with n tip nodes. In this case, the total amount of work needed for all inner nodes is

$$\sum_{i=1}^{n-1}(2i)^2 = 4\frac{(n-1)n(2(n-1)+1)}{2}$$

which reduces to $\mathcal{O}(n^3)$. □

2 Auto-detection of minimum branch length threshold

During phylogenetic inference, the topology of the tree is forced to remain binary at all steps of the optimization in most common implementations. Therefore, very short non-zero branch lengths are enforced among identical sequences to retain the binary shape of the tree. These branch lengths are significantly smaller than the rest, and thus, it is probable that the method falsely classifies these two groups of branch lengths into within and between species processes, respectively. One way to avoid this type of error is by preprocessing the data and keeping one representative per sequence cluster. Such a procedure is trivial when considering identical sequences (for instance, using the "-f c" option in RAxML [5]). However, it turns into an optimization problem when selecting over sequences that are of different size, contain gaps or ambiguities. The problem becomes more complex if we further consider that selecting representative sequences from such clusters (i.e. clusters of sequences that are the same but differ in size/gaps/ambiguities) might result in removing informative sites from the phylogenetic inference. To handle this complication, PTP ignores branch lengths below a certain minimum threshold (0.0001), which, based on empirical observations, were found to correspond to nonsensical branch lengths due to similar sequences in the alignment. The weakness of this approach is that such a threshold may vary among different datasets and phylogenetic inference methods. In the current implementation, we therefore use a novel approach to identify the appropriate minimum branch length threshold for any dataset. The aim of the method is to identify the subtrees of the phylogeny that correspond to sequences of zero P-Distance and set the maximum branch length found in these subtrees as the threshold branch length. This approach identifies the correct threshold while it frees the user from cumbersome data preprocessing. For this step of the analysis, both the phylogenetic tree and the alignment are required. Subtrees exclusively comprising branch lengths smaller or equal to this threshold are subsequently ignored in the delimitation step. Figures 1 and 2, present the branch length distribution of the 24 phylogenies used in the study and the branch length threshold value that was identified by the auto-detection method.

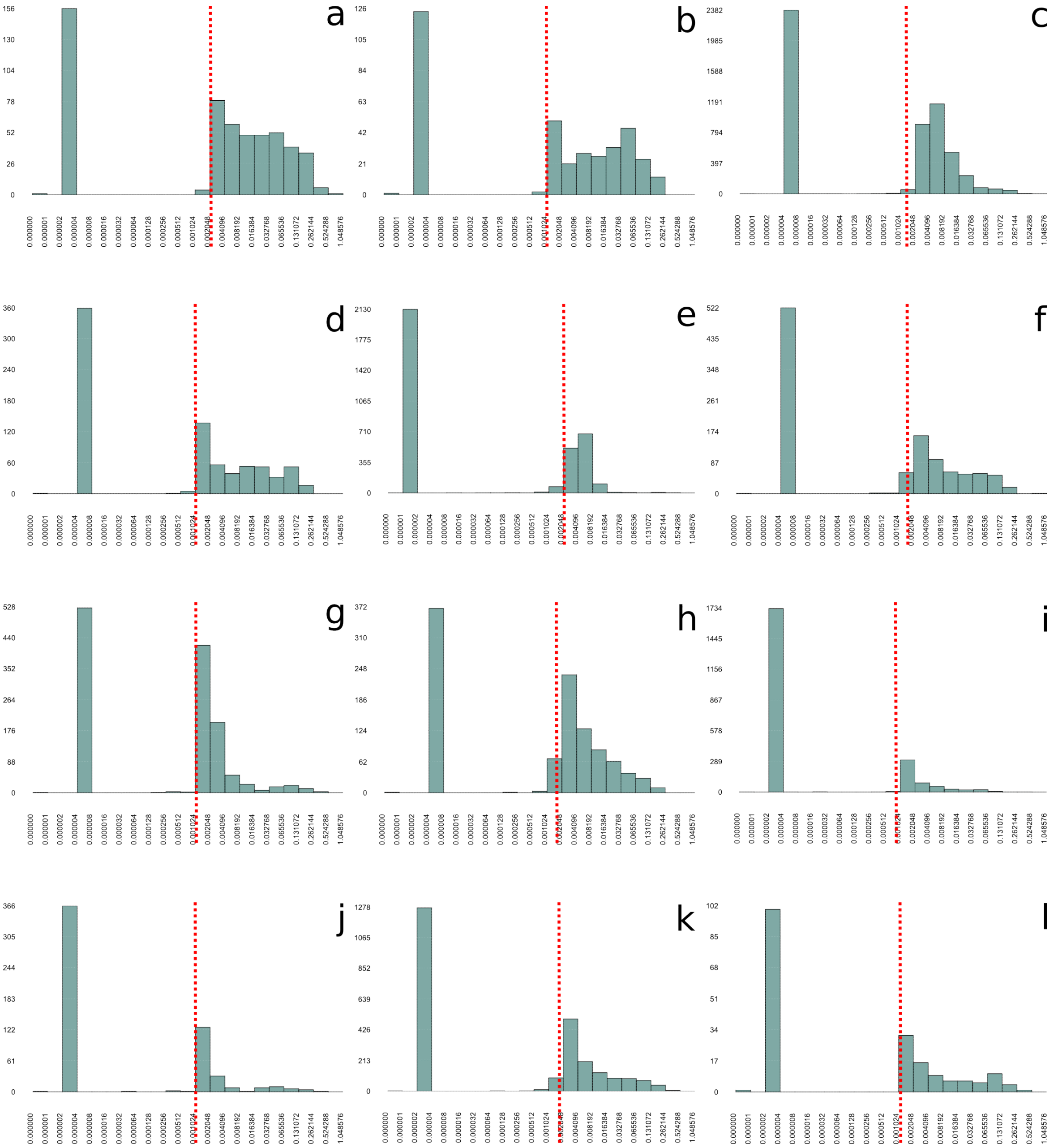

Fig. 1. Branch length distribution of the empirical datasets: a) *Amynthas* b) *Anolis* c) *Anopheles* d) *Atheta* e) *Balanus* f) *Bembidion* g) *Calcinus* h) *Carabus* i) *Clubiona* j) *Coryphopterus* k) *Culicoides* l) *Cyanea*. The y-axis represents the number of sequences and the x-axis the branch lengths. The red dashed line indicates approximately the branch length value under which branch lengths correspond to sequences with a P-Distance of zero

.

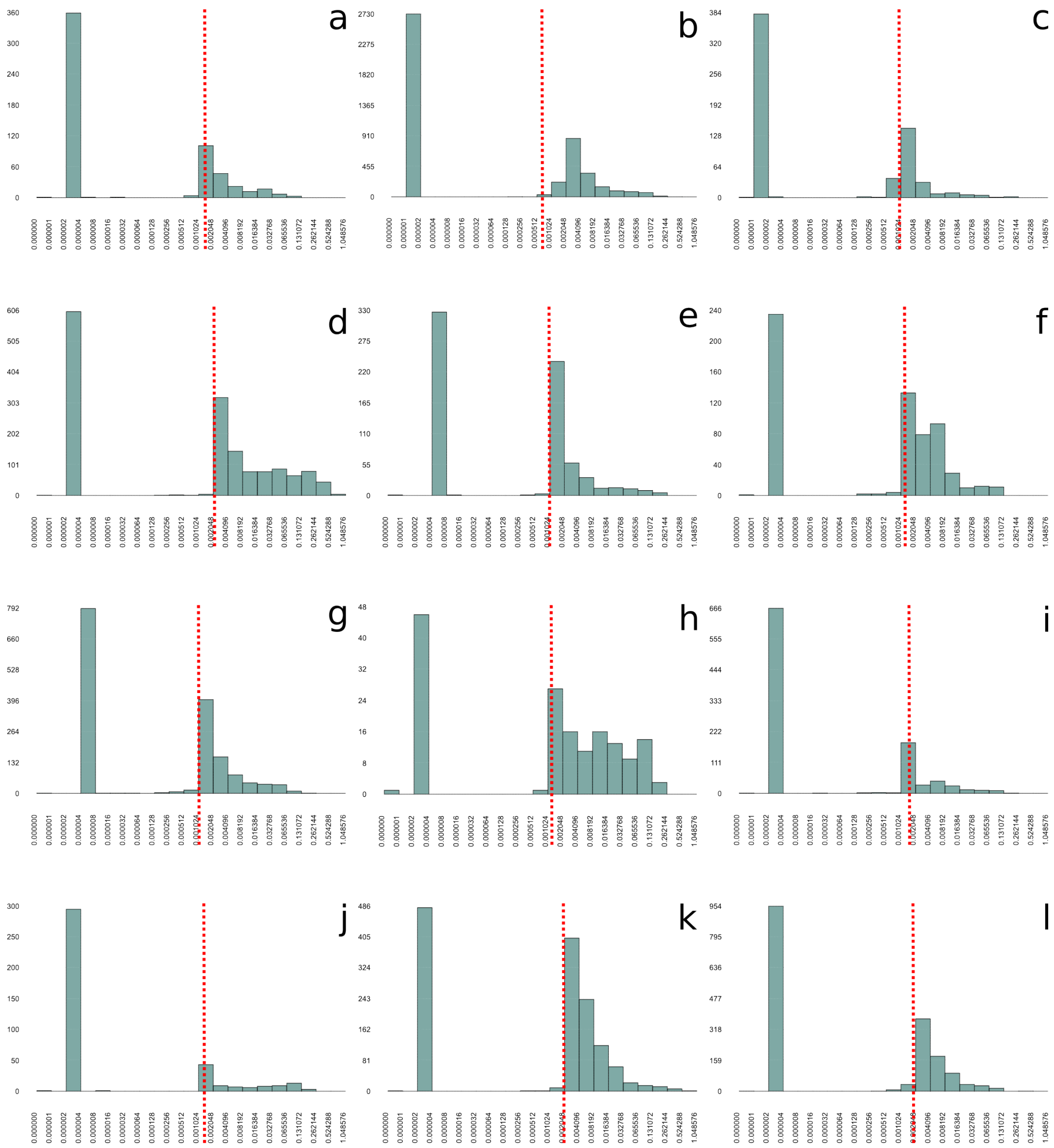

Fig. 2. Branch length distribution of the empirical datasets: a) *Digrammia* b) *Drosophila* c) *Echinococcus* d) *Gammarus* e) *Holothuria* f) *Mopalia* g) *Myotis* h) *Ophiura* i) *Philodromus* j) *Phyllotreta* k) *Rhagada* l) *Xysticus*. The y-axis represents the number of sequences and the x-axis the branch lengths. The red dashed line indicates approximately the branch length value under which branch lengths correspond to sequences with a P-Distance of zero

.

3 Markov Chain Monte Carlo method

Given a tree $T = (V, E)$, the discrete-time MCMC method[1] samples the space of possible delimitations in order to draw *support values* for each node, i.e., the normalized sum of Akaike weights over all sampled delimitations for which a particular node was part of the speciation process.The method explores the space of possible delimitations by proposing a new delimitation from the previous one. This is done by applying with equal probability one of the following two moves:

- **Join.** Join two coalescent processes represented by adjacent subtrees T_v and T_w into a single coalescent process, and set their parent node u (which was part of the speciation process) as the coalescent root.
- **Split.** Split a coalescent process represented by subtree T_u into two new processes represented by subtrees T_v and T_w where v, w are the two direct descendants of u. Node u is now part of the speciation process, and v and w are coalescent roots.

After applying a move, the method computes the maximum likelihood estimator of the rate parameter (rate of exponential distribution) for each coalescent process and for the single speciation process, and then calculates the likelihood of the delimitation.

In the following two subsections, we show that the calculation of the rate parameters is done in $\mathcal{O}(1)$ time for each delimitation, except the initial one which requires $\mathcal{O}(|T|^2)$ time on average. Assuming that the method runs for n MCMC steps, we show that the algorithm requires time $\mathcal{O}(n + |T|^2)$ to compute support values for each node of T, i.e., all subsequent steps (apart from the first step) are independent of the tree size.

3.1 Parameter estimation and computation of log-likelihood

As each possible coalescent process is represented by a subtree of T, we can pre-compute the MLE of the rate parameters for each coalescent process in a single pre-processing step. Recall that the MLE estimate of λ_i for some (sub-)tree $T_i = (E_i, V_i)$ is

$$\hat{\lambda}_i = \left(\frac{1}{|E_i|} \sum_{x \in E_i} \ell(x) \right)^{-1},$$

i.e., $\hat{\lambda}_i$ is dependent on the number of branches of subtree T_i, and the sum of their lengths. Therefore, using a single, bottom-up (postorder) traversal of the tree we can pre-compute the rate parameters for all $|L(T)| - 1$ possible coalescent processes (subtrees) in time $\mathcal{O}(|T|)$. Similarly, we may also pre-compute the likelihood function for each coalescent process (represented by subtree T_i), i.e.,

$$\prod_{x \in E_i} \hat{\lambda}_i e^{-\hat{\lambda}_i \ell(x)}. \tag{6}$$

Due to the exponential number of possible speciation processes [$\mathcal{O}(1.502^m)$ for a tree of m tips, see [7]], we must compute the MLE of its parameter on-the-fly each time we propose a new delimitation. However, it is possible to compute it in $\mathcal{O}(1)$ time if we keep track of the number of branches (*count*) and the sum of their lengths (*sum*) for the speciation process when applying each of the two possible moves:

- **Split.** Sum the lengths of the two edges that were previously part of a coalescent process into *sum*, and increase *count* by two.
- **Join.** Subtract the branch lengths of the two edges that are added to the newly created coalescent process from *sum*, and decrease *count* by two.

[1] A general description of discrete-time MCMC methods appropriate for NP-complete problems can be found in [6].

The likelihood formula (Eq. 6) can be simplified to

$$\hat{\lambda}_i^{|E_i|} e^{-\hat{\lambda}_i \sum_{x \in E_i} \ell(x)}.$$

As it only depends on the number of branches, their sum and the rate parameter, it follows that maintaining the variables *sum* and *count* allows us to compute the likelihood function for the speciation process in $\mathcal{O}(1)$ time.

Similarly, we compute the overall likelihood L' of the new proposed delimitation θ' from the likelihood L of the previous delimitation θ. As a first step, we divide L by the likelihood of the speciation process from θ and multiply it with the likelihood of the new speciation process from θ'. Then, depending on the move used to obtain θ', we perform one of the following operations:

- **Join.** Divide L by the likelihoods of the two coalescent processes from θ (which were joined into a single process in θ'), and multiply it with the likelihood of the new coalescent process.
- **Split.** Divide L by the likelihood of the eradicated coalescent process, and multiply it with the likelihood of the two new coalescent processes.

The new value of L is the likelihood L' of the proposed delimitation. Clearly, it takes $\mathcal{O}(1)$ time to compute the likelihood of each subsequent delimitation from the delimitation of the previous step.

3.2 Support values

The support value of a node u is the normalized sum of Akaike weights [8,9] of all sampled delimitations for which u was part of the speciation process. For simplicity, we will first assume that support values indicate the fraction of sampled delimitations in which a node was part of the speciation process, i.e., we will assume that all sampled delimitations have equal weights. Then, at the end of this section, we will show, with a simple modification, how to accommodate Akaike weights.

Assume *count*(u) stores the number of times (count) node u was part of the speciation process. A naive way to compute it would be to visit all nodes at each MCMC step, and increase *count*(u) by one for every node u that is part of the speciation process. This, however, requires $\mathcal{O}(|T|)$ time and, assuming n MCMC steps, would increase the total run-time of the method to $\mathcal{O}(n|T| + |T|^2)$. We use a faster way that requires an additional variable *start*(u) for each node u, that indicates the MCMC step at which u became part of the speciation process. We further introduce two additional phases; an initialization phase that is executed before the MCMC method (after building the initial delimitation) and a finalization phase that is executed after the MCMC sampling ends.

Initialization step. We visit all nodes and set *start*(u) $= 0$ for each node u that is part of the speciation process in the initial delimitation, and *start*(u) $= -1$ to all nodes which are part of the coalescent process. This step requires $\Theta(|T|)$ time.

Step i of MCMC. At each step i, the MCMC procedure performs one of the following two cases, depending on the type of move that led to the proposed delimitation.

- *Split.* We split a coalescent process with root u into two new coalescent processes and make u part of the speciation process. We set its two direct descendants as new coalescent roots. In case the new proposed delimitation is *accepted*, we set *start*(u) $= i$, i.e., the step from which u started to be part of the speciation process. In the opposite case (*rejected*), we increase *count*(u) by one to account for this one sample in which u was part of a speciation.
- *Join.* We join two coalescent processes with roots v and w into one process with root u (their parent node) making u part of the coalescent process. In case the new proposed delimitation is *rejected*, we decrease *count*(u) by one to account for this one sample in which u was part of a coalescent process. In the opposite case (*accepted*), u will be part of a coalescent process in the next delimitations, and hence we must add to *count*(u) the amount of steps u was in the speciation process since step *start*(u). This is equal to exactly $i -$ *start*(u). Finally, we set *start*(u) $= -1$ to indicate that the node is part of a coalescent process.

Clearly, both cases require $\mathcal{O}(1)$ time.

Finalization step. After the last step of the MCMC method is completed, we visit each node u for which $start(u) \neq -1$ (i.e., nodes that are part of the speciation process) and add to $count(u)$ the number of steps u was in speciation process since $start(u)$, i.e., $n - start(u)$. This requires exactly $\Theta(|T|)$ time.

Therefore, the total run-time of the MCMC method for tree T and n steps is $\mathcal{O}(|T|^2 + n)$ if the maximum likelihood delimitation is used in the initial step, or $\mathcal{O}(|T| + n)$ for a randomly generated initial delimitation.

To accommodate Akaike weights (in short "weights" from now on) in the method, we introduce an additional variable $weights(u)$ for each node u, and a single global variable $weightsum$. We denote the weight of sampled delimitation i as w_i. At step i, the global variable $weightsum$ holds the sum of all sampled delimitations so far, i.e., $\sum_{j=1}^{i} w_j$. Each time a node u becomes part of the speciation process, we set $weights(u)$ to $weightsum$, i.e. the sum of weights of all sampled delimitations so far. The first difference with the previous approach is that whenever a node u becomes part of a coalescent process, we will add to $count(u)$ the difference $weightsum - weights(u)$, which accounts for the sum of weights from the step u became part of the speciation until the current step in which u becomes part of a coalescent. The second difference is that instead of adding a weight of 1 to $count(u)$ at some step i, as described with the assumption of uniform weights, we will have to add w_i.

4 Supplementary Results

4.1 Results of empirical studies

Table 1, presents the efficiency of the five delimitation methods for the 24 empirical datasets used in this study. The efficiency was measured with the three scores: i) percentage of recovered taxonomic species (RTS), ii) F-score and iii) the number of delimited species (for more details refer to the main text).

Table 1. Percentage of RTS, F-scores and number of delimited species for the five delimitation methods (mPTP, PTP, Usearch, Crop and ABGD) for all 24 empirical datasets.

	RTS (%)					F-score					Number of species				
**Genus**	**mPTP**	**PTP**	**Usearch**	**Crop**	**ABGD**	**mPTP**	**PTP**	**Usearch**	**Crop**	**ABGD**	**mPTP**	**PTP**	**Usearch**	**Crop**	**ABGD**
Amynthas	51	40	44	40	44	0.784	0.638	0.649	0.674	0.673	64	104	95	91	90
Phyllotreta	88	69	75	75	75	0.945	0.937	0.944	0.904	0.910	15	18	16	14	17
Atheta	64	60	57	55	60	0.839	0.844	0.836	0.832	0.850	85	100	95	95	91
Philodromus	60	27	47	53	47	0.882	0.717	0.812	0.852	0.828	21	38	26	20	24
Digrammia	70	75	40	50	55	0.901	0.912	0.810	0.751	0.773	26	22	25	16	16
Carabus	35	37	37	41	43	0.699	0.698	0.728	0.727	0.740	42	85	69	62	74
Bembidion	73	74	74	69	72	0.899	0.907	0.914	0.889	0.879	97	115	104	106	97
Calcinus	72	59	69	72	72	0.902	0.836	0.887	0.905	0.897	30	38	42	34	33
Xysticus	74	71	57	51	60	0.929	0.744	0.903	0.882	0.907	40	66	51	48	44
Gammarus	32	38	38	30	36	0.642	0.511	0.486	0.488	0.512	96	177	193	195	177
Clubiona	74	57	74	77	74	0.922	0.777	0.932	0.943	0.930	39	79	39	38	38
Culicoides	59	53	47	43	53	0.828	0.658	0.769	0.768	0.803	132	207	175	197	163
Balanus	75	75	50	50	100	0.964	0.964	0.637	0.935	0.964	5	5	16	7	5
Drosophila	47	51	41	34	53	0.728	0.559	0.747	0.729	0.765	139	444	183	217	157
Anopheles	41	39	30	31	40	0.787	0.704	0.730	0.681	0.730	126	218	193	154	118
Anolis	49	51	51	49	61	0.776	0.765	0.765	0.767	0.844	35	66	66	65	56
Coryphopterus	75	67	42	58	67	0.990	0.986	0.984	0.935	0.990	13	14	16	13	13
Myotis	52	44	48	46	48	0.796	0.793	0.776	0.805	0.811	60	81	74	66	70
Cyanea	60	40	80	80	60	0.763	0.741	0.762	0.781	0.887	11	23	22	17	12
Ophiura	38	31	38	38	38	0.617	0.678	0.667	0.696	0.707	18	22	22	22	20
Holothuria	56	61	67	67	67	0.852	0.858	0.908	0.881	0.883	19	22	22	21	21
Mopalia	55	50	50	45	50	0.932	0.923	0.904	0.854	0.932	20	21	21	23	19
Rhagada	46	42	50	46	42	0.553	0.589	0.445	0.414	0.574	48	71	97	132	50
Echinococcus	71	57	71	57	43	0.934	0.879	0.974	0.893	0.860	9	12	9	10	7

Table 2, presents the same three scores (RTS, F-Score and the number of species) for PTP and mPTP i) when considering all the species of the phylogeny (as in Table 1) and ii) when

considering only the species of the phylogeny that form monophyletic clades. The F-Score, and the recovery of the taxonomic species (RTS) increase substantially when we consider only the monophyletic species, which illustrates the dependency of the methods' accuracy to the proportion of monophyletic species in the phylogeny. On average, the mPTP delimitations were more accurate than PTP, reaching almost optimal inferences if we consider only the monophyletic species (average F-score = 0.945).

Table 2. Percentage of RTS, F-scores and the number of delimited species for PTP and mPTP for all 24 empirical datasets, when considering only the monophyletic species of the phylogeny (mPTP$^\circ$ and PTP$^\circ$) or all of them (mPTP$^\bullet$ and PTP$^\bullet$). In the last row are presented the average RTS, F-scores, and the overall number of delimited species followed by the number of taxonomic species (i.e., average number of species / overall number of taxonomic species or average number of species/number of monophyletic taxonomic species, correspondingly) for each column.

	**RTS (%)**				**F-Score**				**Number of Species**			
Genus	**mPTP$^\bullet$**	**mPTP$^\circ$**	**PTP$^\bullet$**	**PTP$^\circ$**	**mPTP$^\bullet$**	**mPTP$^\circ$**	**PTP$^\bullet$**	**PTP$^\circ$**	**mPTP$^\bullet$**	**mPTP$^\circ$**	**PTP$^\bullet$**	**PTP$^\circ$**
Amynthas	51	79	40	61	0.784	0.915	0.638	0.763	64/43	35/28	104/43	50/28
Phyllotreta	88	100	69	79	0.945	1.000	0.937	0.981	15/16	14/14	18/16	17/14
Atheta	64	88	60	83	0.839	0.978	0.844	0.940	85/84	61/60	100/84	69/60
Philodromus	60	82	27	36	0.882	0.928	0.717	0.701	21/15	20/11	38/15	33/11
Digrammia	70	88	75	88	0.901	0.942	0.912	0.963	26/20	23/17	22/20	19/17
Carabus	35	56	37	53	0.699	0.891	0.698	0.841	42/49	37/34	85/49	60/34
Bembidion	73	93	74	90	0.899	0.966	0.907	0.964	97/97	79/80	115/97	88/80
Calcinus	72	88	59	71	0.902	0.961	0.836	0.865	30/29	24/24	38/29	32/24
Xysticus	74	79	71	76	0.929	0.930	0.744	0.732	40/35	38/33	66/35	59/33
Gammarus	32	70	38	74	0.642	0.877	0.511	0.676	96/53	32/27	177/53	50/27
Clubiona	74	81	57	63	0.922	0.965	0.777	0.836	39/35	38/32	79/35	55/32
Culicoides	59	78	53	70	0.828	0.943	0.658	0.666	132/100	89/76	207/100	149/76
Balanus	75	100	75	100	0.964	1.000	0.964	1.000	5/4	3/3	5/4	3/3
Drosophila	47	65	51	69	0.728	0.818	0.559	0.552	139/148	102/108	444/148	380/108
Anopheles	41	74	39	69	0.787	0.952	0.704	0.919	126/121	75/68	218/121	91/68
Anolis	49	57	51	60	0.776	0.836	0.765	0.781	35/41	33/35	66/41	54/35
Coryphopterus	75	90	67	80	0.990	0.991	0.986	0.986	13/12	11/10	14/12	12/10
Myotis	52	70	44	65	0.796	0.927	0.793	0.877	60/54	40/37	81/54	59/37
Cyanea	60	75	40	50	0.763	1.000	0.730	0.785	11/6	4/4	23/6	8/4
Ophiura	38	75	31	50	0.617	0.915	0.678	0.857	18/13	8/8	22/13	12/8
Holothuria	56	83	61	92	0.852	0.944	0.858	0.949	19/18	11/12	22/18	13/12
Mopalia	55	100	50	91	0.932	1.000	0.923	0.976	20/20	11/11	21/20	12/11
Rhagada	46	92	42	83	0.553	1.000	0.589	0.969	48/24	12/12	71/24	13/12
Echinococcus	71	100	57	80	0.934	1.000	0.879	0.889	9/7	5/5	12/7	6/5
**Average/Sum***	**59**	**82**	**53**	**72**	**0.828**	**0.945**	**0.775**	**0.853**	**$1190^\star/1044^\star$**	**$805^\star/749^\star$**	**$2048^\star/1044^\star$**	**$1344^\star/749^\star$**

In terms of likelihood, when we compared mPTP to PTP for a fixed delimitation, mPTP was always yielding at least as good scores as the PTP but higher in most cases (Table 3). For a fixed delimitation, the single rate is nested to the multi-rate model, hence, they can be compared using the Likelihood Ratio Test (LRT). Table 3, shows that based on the LRT results, the mPTP likelihood was significantly better in all empirical datasets of the study, except for three cases (i.e., *Balanus*, *Digrammia*, and *Mopalia*) for which PTP and mPTP resulted in identical or very similar likelihood scores.

4.2 Execution times

Large biodiversity studies often involve hundreds or thousands of sequences. Computational resources can be a limiting factor for the analysis of such data, and thus, short execution times can be a crucial factor for software selection. The novel dynamic programming implementation of mPTP computes the Maximum Likelihood (ML) delimitation scheme almost instantaneously even for phylogenies involving thousands of sequences. Compared to the original PTP implementation, it is at least five orders or magnitude faster (see Table 4). Comparing the execution times with

Table 3. Likelihood scores of the multi-rate (mPTP) and the single rate (PTP) model for a fixed delimitation (the maximum likelihood delimitation of mPTP). The mPTP likelihood is considered significantly higher according to the LRT (i.e., p-values < 0.05); *df* stands for degrees of freedom (i.e. the difference in the number of free parameters among the two models).

**Genus**	**Likelihood**		**df**	**P-Value**
	**mPTP**	**PTP**		
Amynthas	879.269699	1013.996541	64	<0.05
Anolis	615.66034	719.343417	35	<0.05
Anopheles	11160.505028	11587.360759	126	<0.05
Atheta	1344.728224	1505.603891	85	<0.05
Balanus	5380.243776	5380.385283	7	0.9999
Bembidion	1576.614873	1901.293774	101	<0.05
Calcinus	3427.868124	3541.866781	30	<0.05
Carabus	2214.986697	2445.66532	42	<0.05
Clubiona	2228.954635	2386.287184	39	<0.05
Coryphopterus	774.397645	911.431971	13	<0.05
Culicoides	4098.182625	4365.748878	132	<0.05
Cyanea	272.097349	323.251258	11	<0.05
Digrammia	854.864657	856.717461	27	0.99999997
Drosophila	7193.064555	7809.53444	139	<0.05
Echinococcus	982.254823	995.106962	9	<0.05
Gammarus	1728.829603	2700.411389	96	<0.05
Holothuria	1664.11011	1760.525655	19	<0.05
Mopalia	1377.395058	1385.286695	20	0.730
Myotis	3359.01551	3425.607123	64	<0.05
Ophiura	271.614087	306.487971	19	<0.05
Philodromus	1151.19716	1274.296088	21	<0.05
Phyllotreta	305.128951	335.490926	15	<0.05
Rhagada	3230.016142	3429.410857	48	<0.05
Anolis	615.66034	719.343417	35	<0.05
Xysticus	3035.704608	3081.835973	40	<0.05

the other software used in this study was not as straight-forward, since they differ in the preprocessing of the data [i.e. (m)PTP requires a phylogeny] while for some of them (e.g. Crop, ABGD) the execution time changes drastically with respect to the input parameters.

In Table 5 we further provide the execution times for assessing the confidence of the ML solution using MCMC sampling. For each dataset, we executed ten MCMC runs of 2×10^7 steps each, starting from an initial random delimitation every time. We estimated the convergence of the independent runs by calculating the "Average Standard Deviation of Delimitation Support Values" (ASDDSV), and to obtain an overall support for the ML estimate, we computed the mean "Average Support Value" (ASV) over all ten independent runs. Comparing the equivalent execution times with the MCMC implementation of PTP [10] (available at `http://species.h-its.org/`) is impossible, considering the execution time for the ML optimization alone.

	**Execution Time**	
**Genus**	**PTP**	**mPTP**
Amynthas	0m27.068s	0m0.013s
Phyllotreta	0m8.840s	0m0.007s
Atheta	2m10.569s	0m0.019s
Philodromus	4m9.498s	0m0.016s
Digrammia	0m57.262s	0m0.008s
Carabus	7m20.678s	0m0.040s
Bembidion	11m9.534s	0m0.029s
Calcinus	9m56.954s	0m0.051s
Xysticus	23m58.893s	0m0.055s
Gammarus	70m13.939s	0m0.069s
Clubiona	85m49.569s	0m0.036s
Culicoides	143m0.667s	0m0.125s
Balanus	198m17.230s	0m0.187s
Drosophila	844m32.892s	0m0.287s
Anopheles	2031m3.358s	0m0.733s
Anolis	0m8.983s	0m0.007s
Coryphopterus	0m30.269s	0m0.010s
Myotis	48m53.366s	0m0.056s
Cyanea	0m7.573s	0m0.003s
Ophiura	0m1.952s	0m0.003s
Holothuria	1m14.270s	0m0.016s
Mopalia	0m58.813s	0m0.016s
Rhagada	15m25.988s	0m0.066s
Echinococcus	1m2.249s	0m0.010s

Table 4. Execution times for the estimation of the inference of the ML delimitation with PTP and mPTP for each of the 24 empirical datasets

Genus	ASDDSV	ML ASV (%)	Execution Time
Phyllotreta	0.001991	72	0m38.588s
Atheta	0.000605	92	0m49.332s
Philodromus	0.003614	92	0m59.615s
Digrammia	0.001561	86	1m4.019s
Carabus	0.001857	86	1m3.661s
Bembidion	0.000867	91	0m56.494s
Calcinus	0.000079	99	0m55.099s
Xysticus	0.00742	79	0m55.573s
Gammarus	0.000548	91	0m56.498s
Clubiona	0.003026	83	0m54.260s
Culicoides	0.003973	94	0m59.164s
Balanus	0.000004	100	0m47.911s
Drosophila	0.048099	88	1m2.911s
Anopheles	0.016768	92	1m9.379s
Amynthas	0.000564	88	0m54.368s
Cyanea	0.000175	91	1m4.829s
Ophiura	0.000299	84	1m1.562s
Holothuria	0.00017	99	0m54.739s
Mopalia	0.002167	89	0m56.258s
Rhagada	0.005595	85	0m58.763s
Echinococcus	0.000248	95	0m54.002s
Anolis	0.000415	82	0m54.772s
Coryphopterus	0.000613	97	0m54.055s
Myotis	0.002721	89	0m57.154s

Table 5. The table shows the results for 1) the ASDDSV, 2) the ASV for the ML delimitation and 3) the accumulated execution time for 10 independent MCMC runs with 2×10^7 MCMC steps per run, for each of the datasets.

References

1. E.W. Weisstein. *CRC Concise Encyclopedia of Mathematics, Second Edition*. CRC Press, 2002.
2. R.L. Graham, D.E. Knuth, and O. Patashnik. *Concrete Mathematics: A Foundation for Computer Science*. A foundation for computer science. Addison-Wesley, 1994.
3. Sloane N.J.A. and Wolfdieter Lang. Sequence A000346. `https://oeis.org/A000346`.
4. P. Flajolet and R. Sedgewick. *Analytic Combinatorics*. Cambridge University Press, 2009.
5. Alexandros Stamatakis. RAxML version 8: a tool for phylogenetic analysis and post-analysis of large phylogenies. *Bioinformatics*, page btu033, 2014.
6. Mark Jerrum and Alistair Sinclair. The Markov Chain Monte Carlo method: an approach to approximate counting and integration. In *Approximation Algorithms for NP-hard Problems*. PWS Publishing Co., Boston, MA, USA, 1997.
7. Tomochika Fujisawa and Timothy G. Barraclough. Delimiting Species Using Single-Locus Data and the Generalized Mixed Yule Coalescent Approach: A Revised Method and Evaluation on Simulated Data Sets. *Systematic Biology*, 62(5):707–724, 2013.
8. Hirotugu Akaike. A Bayesian analysis of the minimum AIC procedure. *Annals of the Institute of Statistical Mathematics*, 30(1):9–14, 1978.
9. KP Burnham and DR Anderson. Model Selection and MultiModel Inference: A Practical Information-Theoretic Approach, 2002.
10. Jiajie Zhang. *Models and algorithms for phylogenetic marker analysis*. PhD thesis, Lübeck, Univ., Diss., 2015, 2015.
